# When to use next generation sequencing or diagnostic PCR in diet analyses

**DOI:** 10.1111/1755-0998.12974

**Published:** 2019-02-04

**Authors:** Oskar Rennstam Rubbmark, Daniela Sint, Sandra Cupic, Michael Traugott

**Affiliations:** ^1^ Mountain Agriculture Research Unit, Institute of Ecology University of Innsbruck Innsbruck Austria

**Keywords:** DNA barcoding, food web, high‐throughput sequencing, metabarcoding, multiplex PCR, prey detection, trophic interactions

## Abstract

Next‐generation sequencing (NGS) is increasingly used for diet analyses; however, it may not always describe diet samples well. A reason for this is that diet samples contain mixtures of food DNA in different amounts as well as consumer DNA which can reduce the food DNA characterized. Because of this, detections will depend on the relative amount and identity of each type of DNA. For such samples, diagnostic PCR will most likely give more reliable results, as detection probability is only marginally dependent on other copresent DNA. We investigated the reliability of each method to test (a) whether predatory beetle regurgitates, supposed to be low in consumer DNA, allow to retrieve prey sequences using general barcoding primers that co‐amplify the consumer DNA, and (b) to assess the sequencing depth or replication needed for NGS and diagnostic PCR to give stable results. When consumer DNA is co‐amplified, NGS is better suited to discover the range of possible prey, than for comparing co‐occurrences of diet species between samples, as retested samples were repeatedly different in prey detections with this approach. This shows that samples were incompletely described, as prey detected by diagnostic PCR frequently were missed by NGS. As the sequencing depth needed to reliably describe the diet in such samples becomes very high, the cost‐efficiency and reliability of diagnostic PCR make diagnostic PCR better suited for testing large sample‐sets. Especially if the targeted prey taxa are thought to be of ecological importance, as diagnostic PCR gave more nested and consistent results in repeated testing of the same sample.

## 1 INTRODUCTION

DNA‐based diet analyses are increasingly being used to track feeding interactions in various ecosystems (Pompanon et al., 2012; Traugott, Kamenova, Ruess, Seeber, & Plantegenest, 2013; Symondson & Harwood, 2014; Clare et al., 2011). Early on, DNA‐based prey detections were usually targeting single or a set of prey species using singleplex or multiplex PCR assays (e.g., Harper et al., 2005; Juen & Traugott, 2007; Szendrei, Greenstone, Payton, & Weber, 2010). Using this approach, diet samples such as regurgitates, faecal samples or whole body extracts have been tested for diet items that “a priori” have been deemed interesting (e.g., King et al., 2011, Wallinger et al., 2012, Staudacher, Jonsson, & Traugott, 2016). With advances in next‐generation sequencing (NGS) platforms, such an a priori selection of targeted diet items is no longer needed. NGS‐based metabarcoding instead relies on general primers to amplify the DNA from as many of the species contained within diet samples as possible. This bulk amplified DNA is sequenced using NGS, producing millions of sequences that are then matched to reference databases (Pompanon et al, 2012).

The main benefit of NGS is that, in theory, this method has the potential to reveal the identity of all of the DNA contained in samples, and therefore, NGS is now more and more being used in both diet analyses (e.g., Deagle, Kirkwood, & Jarman, 2009; De Barba et al., 2014; Crisol‐Martínez et al., 2016; Salinas‐Ramos et al., 2015; Sousa et al., 2016; Vesterinen et al., 2016) and detection of species from environmental DNA (eDNA) (e.g., Taberlet, Coissac, Hajibabaei, & Rieseberg, 2012; Bohmann et al., 2014; Shaw, Weyrich, & Cooper, 2017). In reality, however, describing the complete contents of samples is difficult, as shown by several studies that sequenced mock‐samples and failed to detect many of the included species (e.g., Leray et al, 2013; Clarke, Soubrier, Weyrich, & Cooper, 2014; Elbrecht & Leese, 2015; Rennstam Rubbmark, Sint, Horngacher, & Traugott, 2018). There are several reasons why species failed to be detected in these samples, including a preferential amplification of certain DNA templates by the used primers (Elbrecht & Leese, 2015; Rennstam Rubbmark et al., 2018), or because proportions of different DNA types were unbalanced in the tested sample and drastically affected the probability of detecting lower concentration DNA types (Rennstam Rubbmark et al., 2018). In addition, even for detected taxa there is some variability expected in how likely different DNA templates are to be detected. For example, the relative abundances of sequences are not always proportional to tissue mass included in (dietary) samples.

For these reasons, it has been stressed that in NGS metabarcoding data sequence numbers can be misleading (Yu et al., 2012; Deagle, Thomas, Shaffer, Trites, & Jarman, 2013, Elbrecht & Leese, 2015; Ficetola et al., 2015), and because of this, even though NGS metabarcoding data may have quantitative aspects, it is usually treated as incidence/qualitative data (Deagle et al., 2018). However, sequencing depth (a proxy for sampling effort in NGS) still needs to be high enough to reliably characterize samples. This is especially important in diet samples, where varying type or amounts of tissue may have been consumed depending on prey type. However, despite this no clear recommendation for the level of sequencing that is required currently exists. Recent studies have aimed at a mean read number per sample that in a few cases has been as high as ~500,000, but more frequently has been in the ~50,000 to ~1,000 range (see Appendix S3 for a review of mean reported sequencing depths per samples for diet sequenced on the MiSeq platform). In addition, it has been reported that sample read numbers can vary strongly from the targeted read number (e.g., Briem et al., 2018).

This is problematic, for example, when comparing alpha or beta diversity, where an unequal sampling effort or completeness across samples will induce biases and give misleading results as prey taxa that are present in samples may be missed purely by chance (Soberón & Llorente, 1993; Gotelli & Colwell, 2001; Cardoso, Borges, & Veech, 2009). Paradoxically, when sequencing depth is increased, it has been reported that this also will inflate the presence of erroneous and unwanted reads (Alberdi, Aizpurua, Gilbert, & Bohmann, 2017). For diet analyses, this is problematic as the DNA in diet samples (a) often will be dominated by unwanted consumer DNA and (b) may contain very different proportions of prey DNA depending on how much/recently each prey type was consumed. The problem is thus threefold where (a) consumer DNA will produce a larger number of reads than prey. (b) sequencing depth of diet reads needs to be sufficient to compensate for unequal detection probabilities in diet samples. (c) The sequencing depth that would be needed to achieve this is usually not known and will depend on the relative proportions between DNA types included in samples.

While researchers are often aware of such problems, their magnitude, what this means for the reliability of results, and how this can be compensated for have so far not been well addressed. One way of approaching such problems would be to first select diet samples, known to be low in consumer DNA, such as regurgitates or faeces (Raso et al., 2014; Waldner & Traugott, 2012), and compensate with more sequencing depth to increase diet‐reads, or replicate PCRs to increase the reliability in the taxa detected (Alberdi et al., 2017). This would increase the sampling intensity of diet reads; however, the amount of sequencing that is needed to consistently describe the content of such samples still needs to be investigated to show if this is a realistic approach. Alternatively, for a similar effect, the amplification of consumer DNA could be reduced (see O'Rorke et al., 2012 for a review). Among methods that allow this is the use of less general primers that do not amplify predator DNA (e.g., Deagle et al., 2007; Vesterinen et al., 2016), or the use of blocking primers to prevent primers from amplifying predator DNA (Vestheim & Jarman, 2008). The latter has been attempted for a range of studies (e.g., Deagle et al., 2009; Deagle, Chiaradia, McInnes, & Jarman, 2010; Sousa et al., 2016), however, not always successfully (e.g., Maghsoud et al., 2014; Gomez‐Polo et al., 2016). The main problem with such methods is that preventing the amplification of consumer DNA in many cases is not possible or also excludes certain prey types. This is especially problematic when the DNA of the consumer is not sufficiently different from all likely prey DNA to enable the blocking primers to be designed (O'Rorke et al., 2012). Even when possible, this limits the range of predators that can be realistically investigated, as specific primers would have to be designed and tested for all investigated predator and many potential prey species to ensure they work as desired.

In contrast to NGS metabarcoding, diagnostic PCR does not allow the DNA from more than a few types of targeted diet items to be identified, even if primers are being multiplexed. This method is also purely qualitative, meaning that it offers little possibility to quantify the proportions between DNA types contained within samples. Despite these drawbacks, the benefit of a multiplex PCR detection system is that its sensitivity can be bench‐marked so that the number of template copies that are needed for successful amplification is known and equalized for each included primer pair (Sint, Raso, & Traugott, 2012). Diagnostic PCR is often employed qualitatively to simply test for presence/absence of specific prey taxa, although it also offers the possibility to estimate the quantity of prey DNA contained within diet samples by applying capillary electrophoresis or qPCR. When the DNA content in a sample is low enough that the threshold for detectability is approached, the stochastic nature of PCR will cause some variation in detection success even with this method, typically close to the threshold of detection (e.g., Sint, Raso, Kaufmann, & Traugott, 2011). This approach is, however, considerably cheaper and allows thousands of individual samples to be checked for the targeted food species, which would make this variability less likely to affect the overall conclusions. Furthermore, as variance around the threshold of detection is likely to be attributable to measurement errors or variation within PCR efficiency, the relatively low cost per sample allows detections below a certain threshold to be retested to improve the reliability of results.

Here, we want to specifically investigate how (a) NGS metabarcoding data compares to diagnostic multiplex PCR in how well it describes the contents of predatory beetle regurgitate samples, in repeated testing of the same sample. Specifically, we aim to identify what replication level or sequencing depth would be needed, to attain a reliable description of prey DNA contained in beetle regurgitates using each method. The rationale here is that, even if we are not able to describe the complete content of a sample, the subset of DNA that is described from each sample at least needs to be drawn in a way that gives the same result in repeated testing of the same sample. If this is not the case, samples will not be well described and a sensible comparison of co‐occurrences of diet items between sets of samples will be difficult. In addition, we here specifically selected regurgitate samples to test whether these are low enough in consumer DNA that they could be NGS metabarcoded without the need to block consumer DNA. We then opted to increase sequencing depth for NGS, to test (b) how much sequencing this type of diet sample requires, when sequenced on an NGS platform without blocking primers (Vestheim & Jarman, 2008).

## 2 MATERIALS AND METHODS

### 2.1 Samples

For this study, a subset of 20 regurgitates (10 per taxon) collected in an ongoing study were selected from *Poecilus cupreus *(Carabidae) and *Philonthus* sp. (Staphylinidae) (for a detailed sampling description see Appendix S1).

### 2.2 DNA extraction

Each regurgitate was extracted using a Biosprint96® robotic platform with the Biosprint 96 DNA Blood Kit (Qiagen, Hilden, Germany) according to the manufacturer's recommendations, except that buffer ATL was replaced by TES buffer (0.1 M TRIS, 10 mM EDTA, 2% SDS; pH 8) for lysis (>12 hr at 56°C) and 200 µl 1× TE buffer was used for elution.

### 2.3 Library preparation

Library preparation for NGS followed a “single tube PCR” approach, described in Rennstam Rubbmark et al. (2018) that allows to amplify the desired DNA fragment, add molecular identifiers (MID) and adapters for the MiSeq platform (Illumina, San Diego, USA) in a single reaction (detailed protocol and description in Appendix S2). The primers used to do this have previously been used for library preparation and successful sequencing (Rennstam Rubbmark et al., 2018), and amplify a fragment of ~313 bp of the cytochrome *c* oxidase subunit I (COI). Using this approach, each regurgitate sample was subjected to six replicated PCRs (detailed protocol and description in Appendix S2). Each of the 120 PCR products was labelled with a unique MID‐combination and combined into a ready‐to‐sequence library. For this library, PCR products were first quantified using the QIAxcel advanced (Qiagen) capillary electrophoresis system with the software ScreenGel v1.4 (Qiagen). sequencing depth was then adjusted between samples by including them at different concentrations in order to make sure that (a) half of the expected 20 M reads from a full MiSeq (Illumina, San Digeo, USA) run would be assigned to the high replicate reads (one replicate per sample; 500,000 reads per replicate; Box 1), whereas the remaining half would be assigned to (ii) low depth replicates (five replicates per sample; 100,000 reads per replicate; Box 1), in order to give each sample a total of 1 M reads split between replicates. These levels were set based on recently published studies (see Appendix S3 for a review of mean reported sequencing depths per samples for diet sequenced on the MiSeq platform). To adjust sequencing depth, first five replicates of each sample were pooled equimolarly, and then, this pool was mixed equimolarly with the 6th replicate (Box 1). This was done to allow us to investigate the effect of sequencing depth on the recovery of species. After pooling, samples were cleaned from fragments smaller than amplicon size using SPRIselect (Beckman Coulter, Bread, USA) according to the manufacturer's recommendation for left side size selection and with a ratio of 0.8 of beads to PCR product volume. After clean‐up, the DNA was quantified with the QIAxcel advanced system and all 20 samples were pooled at equimolar concentrations into a library that was submitted along with custom sequencing primers (Rennstam Rubbmark et al., 2018) for sequencing on an Illumina MiSeq platform using the MiSeq Reagent Kit v2 (250 bp, Illumina, San Digeo, USA) at the Biomedical Sequencing Facility of the CeMM Research Center for Molecular Medicine of the Austrian Academy of Sciences and the Medical University of Vienna.

BOX 1 **Graphical representation of the sample pooling that was done during NGS library preparation in order to adjust sequencing level for “high” and “low” sequencing depth samples [Colour figure can be viewed at wileyonlinelibrary.com]**

1

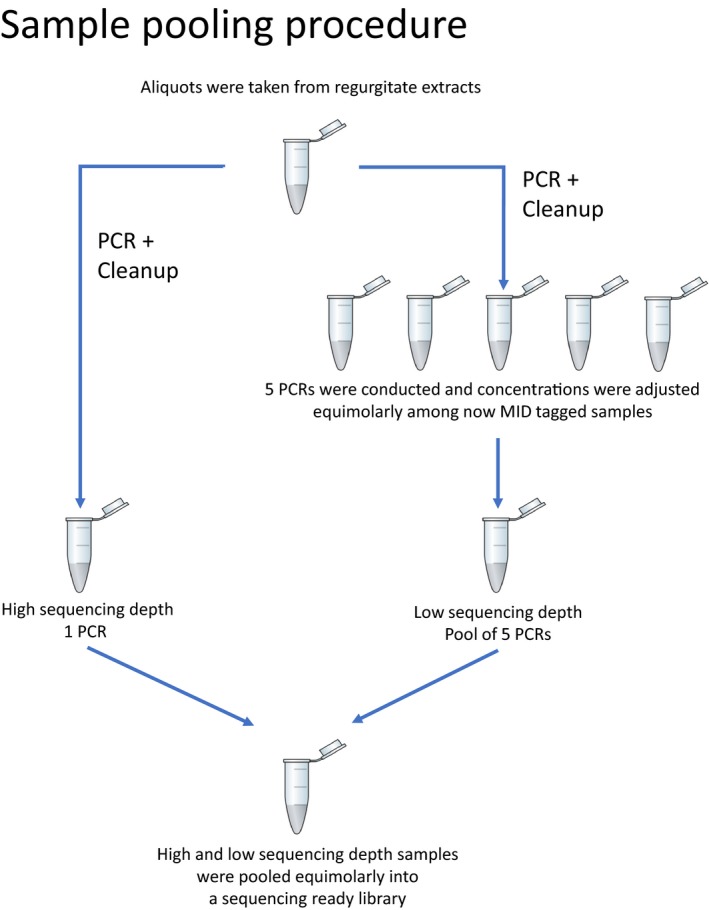

### 2.4 Multiplex PCR assay

To compare how consistent the detection of species targeted by diagnostic PCR was between replicated PCRs, we assembled a set of published and newly designed primers in a multiplex detection system. This multiplex detection system was designed to target the consumption of four species of aphids (*Acyrthosiphon pisum*, *Metopolophium dirhodum*, *Rhopalosiphum padi* and *Sitobion avenae*), Collembola, Lumbricidae, as well as the Cereal Leaf beetle *Oulema melanopus* (Table 1). It was originally designed for another study in order to test how the availability of decomposer prey affects the consumption of pest prey in an agricultural setting (i.e., targeting functionally important prey types).

**Table 1:** Primers used to screen for targeted prey taxa in the multiplex PCR assay. Provided are the targeted taxa/species, the primer name, the primers’ sequences, the fragment length amplified by each prime pair, the targeted gene, the final concentration (Conc.) of each primer in the PCR and the references where primers have first been described

Target group	Primer	Sequence (5′−3′)	Fragment (bp)	Gene	Conc. (μM)	References
Lumbricidae	S408‐earthw	CCATGATTTCTTAGATCGTACAATCC	85	18s	0.8	Staudacher et al. (2016)
	A413‐earthw	ATARGGGTCGGAGCTTTGTG				Staudacher et al. (2016)
Collembola	Col3F	GGACGATYTTRTTRGTTCG	231	18s	0.2	Kuusk & Agusti (2008)
	A415‐springt	GAATTTCACCTCTAACGTCGCAG				Staudacher et al. (2016)
*Acyrthosiphon pisum*	Acy‐pis‐S492	GTCCTGATATATCATTTCCTCGC	210	COI	0.08	This study
	Acy‐pis‐A496	AAATTGATGAAATTCCTGCTAGG				This study
*Metopolophium dirhodum*	Met‐dir‐S436	CCTTTATCAAATAACATTGCACATAAC	105	COI	0.4	Ye et al. (2017)
	Met‐dir‐A440	AATAAAGTTAATTGCTCCTAAAATTGAG				Ye et al. (2017)
*Rhopalosiphum padi*	Rho‐pad‐S440	TAATAATATAAAATTAAACCAAATTCCATTA	136	COI	0.3	Ye et al. (2017)
	Rho‐pad‐A442	TGATGTATTTAAATTACGATCAGTAAGAAG				Ye et al. (2017)
*Sitobion avenae*	Sit‐ave‐S433	TCATCACTTAGAATTCTTATTCGTCTT	304	COI	0.1	Ye et al. (2017)
	Sit‐ave‐A438	AAGGTGGRTAAATAGTTCATCCTGTA				Ye et al. (2017)
*Oulema melanopus*	Om‐S2‐KS‐S185	TTGACTTCTCCCACCTTCAA	248	COI	0.2	This study
	Om‐A‐KS‐A184	CAAACAGAGGCATTCGATCT				This study

### 2.5 Development of the assay

Concentration of individual primer pairs (Table 1) was optimized based on standardized DNA templates as described in Sint et al. (2012) to minimize biases in detections between amplified prey targets. To do this, a sensitivity test was first conducted to identify the detection limit for each target. This test showed that in a mix of extracted DNA from all targeted species, amplification was most efficient at a concentration of more than 1,200 double‐stranded copies of template DNA. At lower levels than this, signal strength (measured as relative fluorescent units [RFU]) began to weaken. In testing towards a single target species, amplification was, however, more efficient, and sensitivity tests showed that amplification was efficient until a concentration of less than 150 double‐stranded copies of template DNA.

To confirm that no nonspecific amplification was occurring, all target‐specific primers were tested on a set of 122 representatives of nontarget species known to commonly occur in cereal fields (Appendix S4). Furthermore, a subset of detections from field samples was sequenced to confirm the correct identity of detected targets.

### 2.6 Final protocol

Based on this testing, PCRs were performed in a 10 µl reaction mix containing 2.5 µl DNA extract, 0.5 µl BSA (10 mg/ml), 1 µl of primer mix (Table 1), 5 µl KAPA2G Fast Multiplex Mix (Peqlab, Erlangen, Germany) and 1 µl PCR grade water. Cycling conditions on a Mastercycler Nexus (Eppendorf, Germany) were set to 3 min at 95°C, 35 cycles of 15 s at 95°C, 90 s at 62.5°C, 30 s at 72°C and final elongation for 10 min at 72°C. Amplification of all PCR products was verified using the QIAxcel advanced system (version 3.2; Method: AL320; Qiagen) together with the Qiaxcel DNA Screening Gel Cartridge (Qiagen). A successful amplification of each respective target was defined as a DNA fragment of the expected length with a corresponding signal strength of ≥0.07 relative fluorescent units (RFU). Using this diagnostic multiplex PCR assay, each regurgitate sample was subjected to five replicated PCRs.

### 2.7 Analyses of NGS metabarcoding data

Raw sequencing reads were demultiplexed, quality checked, trimmed and combined into paired‐end reads using Usearch with a maximum of 80 mismatches allowed (Edgar, 2010). Reads were then dereplicated using Usearch to remove reads shorter than 300 bp. Remaining reads were clustered into molecular operational taxonomic units (MOTUs) based on a 97% sequence similarity using Usearch. From each cluster, the centroid sequence was selected as representative, and for each of these sequences, taxonomic IDs were assigned using blastn with a word size of 28 bp (Altschul, Gish, Miller, Myers, & Lipman, 1990) based on the NCBI nucleotide database (Benson, Karsch‐Mizrachi, Lipman, Ostell, & Wheeler, 2006). From returned hits, the most likely identity was selected from hits (E‐score cut‐off value: 1e‐10) with a minimum match length of 150 bp and a minimum percentage identity of 90%.

### 2.8 Statistical analysis

All analyses were based on the presence or absence of either amplification of the expected DNA fragment (diagnostic PCR) or retrieved sequences (NGS). A considerable proportion of NGS‐reads did, for all replicates and samples, belong to the same species as the predator that had produced the regurgitate (i.e., the consumer). After a comparison of the difference in the proportion of consumer reads per replicate and sample, all consumer reads were removed from subsequent analyses.

For the regurgitate samples showing at least one positive detection among replicates per prey species in multiplex PCR, the probability of detecting each of the prey species was modelled against the number of tested replicates for multiplex PCR (resampled with replacement from within replicated PCRs). We expected that the reliability of detecting prey DNA within a sample should depend on its amount in the DNA extract and be reflected by the amount of amplicon generated. The RFU value here provides a measure of this on the QIAxcel system that was used to screen samples. However, this correlation is best when the PCR plateau phase is not reached as is typically the case for low‐concentration prey DNA (Griesbach‐Hobbach, 2016). In addition, we expected that the largest amount of variation should occur around the threshold of detection (Sint et al., 2011) due to a certain variability in PCR efficiency and measurement accuracy. Thus, models were fitted separately for samples scoring weak detections (RFU < 0.1) and strong detections (RFU > 0.1). For NGS, the probability of detecting each of the prey species was modelled against sequencing depth and Shannon diversity. For both methods, this was done using Bayesian generalized linear models fitted with binomial error distributions available through the R package “arm” (Gelman & Su, [Link]). We did so in order to estimate how much replication/sampling would have been needed to achieve comparable results with both methods given the specific contents of our samples (as these results depend on the contents of samples they could be different for samples collected from other species or in a different setting). Not all species targeted by multiplex PCR could be recovered with NGS; consequently, the results are only presented for those prey groups that were targeted with diagnostic PCR that also recurred among returned reads.

To contrast the information content described by the two methods, we calculated the Shannon entropy for each replicated sample and method. This was done to give a quantitative measure of the amount of information that can be derived from each sample using either NGS or diagnostic PCR. As ecological questions often require compositional differences among diversity to be compared, we characterized the variability in how species are detected with each method. We did so by calculating how the variability between replicates of the same sample (beta diversity) was split between nestedness (species are gained or lost, but resemble subsets of the most species‐rich samples) and species turnover (species are replaced by different species). To allow multiple samples to be compared, the function “beta.sample()” available from the R package “betapart” (Baselga et al., 2017) was used with 9,999 permutations. Differences between calculated indexes and methods were subsequently tested using linear mixed effect models fitted with binomial error distributions using the R package “lme4” (Bates, Maechler, Bolker, & Walker, 2015). For each model, diagnostic plots were examined to confirm that model assumptions were met (Zuur, Ieno, & Elphick, 2010). All statistical tests were performed in R version 3.3.3 (R Core Team, 2017).

## 3 RESULTS

### 3.1 NGS metabarcoding

A total number of 13,880,320 raw paired‐end reads were generated from a run on the MiSeq platform. After preprocessing, read numbers varied considerably between samples and were distributed between replicated samples with a mean read number of 499,543 reads (*SD*: 353,999) for high sequencing depth samples and 56,193 (*SD*: 43,330) for low sequencing depth samples. One of the *P. cupreus* regurgitates produced exclusively consumer reads in all replicates, and two additional *P. cupreus* regurgitates produced only fungal and bacterial reads outside of consumer reads. As no diet‐reads could be identified, these three samples were subsequently excluded. The proportion of consumer DNA differed between *P. cupreus* (90% at high sequencing depth; 88% at low sequencing depth) and *Philonthus* sp. (87% at high sequencing depth; 81% at low sequencing depth) (*p* < 0.001), and when sequencing depth was increased, the proportion of consumer reads to prey reads increased (*p* < 0.001).

### 3.2 Diagnostic PCR

**Figure 1:**
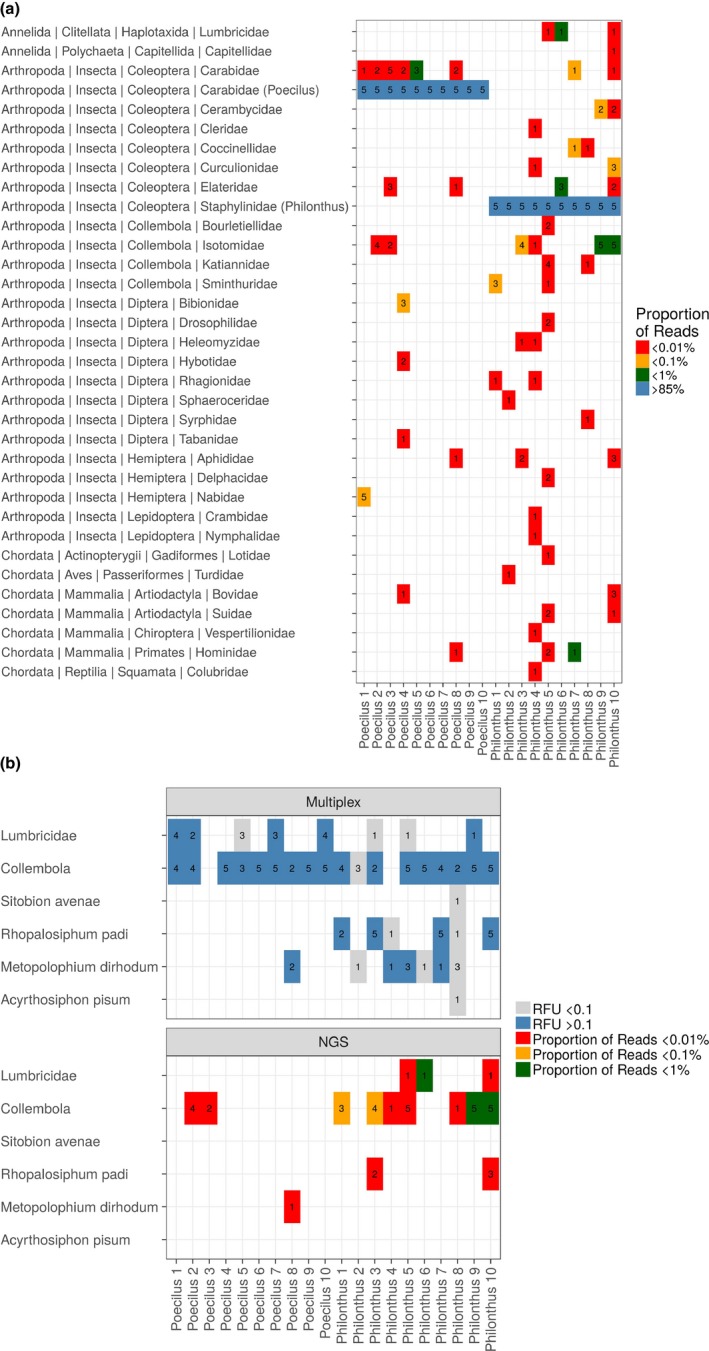
(a) Taxa where DNA was detected from regurgitates of carabid (*Poecilus*) and staphylinid (*Philonthus*) beetles using NGS. Numbers indicate in how many of the five replicates of a sample the respective DNA‐type was detected. Colour is proportional to the relative number of sequences these detections represented from among all reads within a sample. (b) Number of the five replicates of each regurgitate sample that showed detections of prey taxa targeted by multiplex PCR (upper panel) and the detection of these prey taxa using NGS (lower panel). Colour is proportional to the average RFU (detection strength) measured between replicates per group targeted by multiplex PCR and sample [Colour figure can be viewed at wileyonlinelibrary.com]

**Figure 2:**
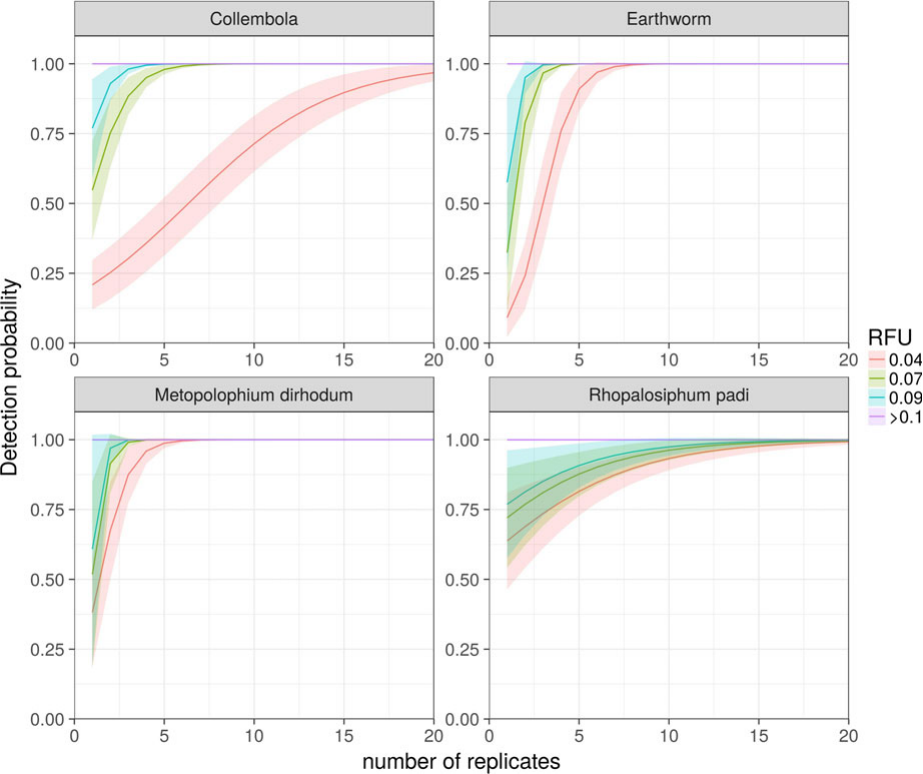
Probability of detecting DNA of specific prey taxa using multiplex PCR by repetitive testing of the same sample. Colour and line type show that this effect varies with detection strength (RFU value). Width of colours represents predicted standard error. Results are predicted from within samples where at least one replicate was confirmed to contain the targeted DNA (*y*‐axis) and are modelled against number of replicated PCRs of the same sample (*x*‐axis). Note that these figures do not aim to show the general level of replication needed for detection of prey DNA, but the amount needed given the DNA of the respective prey type that was contained in each tested regurgitate sample [Colour figure can be viewed at wileyonlinelibrary.com]

Among the taxonomic groups targeted by the multiplex PCR assay, the most commonly detected prey were Collembola, detected in 77% of all replicates, followed by *R. padi* (20%), Lumbricidae (20%), *M. dirhodum* (12%), *S. avenae* (1%) and *A. pisum* (1%), whereas *O. melanopus* was never detected. For all targeted prey, replicated PCRs repeatedly gave different outcomes, however mostly when the measured signal strength was below 0.1 RFU and close to the threshold for a positive detection (0.07 RFU). Detections were more stable across replicates if the signal strength exceeded 0.1 RFU (Figure 1b). However, even though the probability of detecting a prey group (given that it was known to be contained within a sample) was not absolute and depended on the measured RFU value among low signal strength samples, detection probability increased with the level of replication (Figure 2, *p*_Lumbricidae_ = 0.051, *p*_Collembola_ < 0.001, *p*_R.padi_ < 0.05). Additionally, for both Lumbricidae and Collembola prey, detection probability increased more strongly with an increased replication level for lower RFU values (Figure 2, *p*_Lumbricidae_ < 0.001, *p*_Collembola_ < 0.001).

### 3.3 Comparison between NGS metabarcoding and diagnostic multiplex PCR

**Figure 3:**
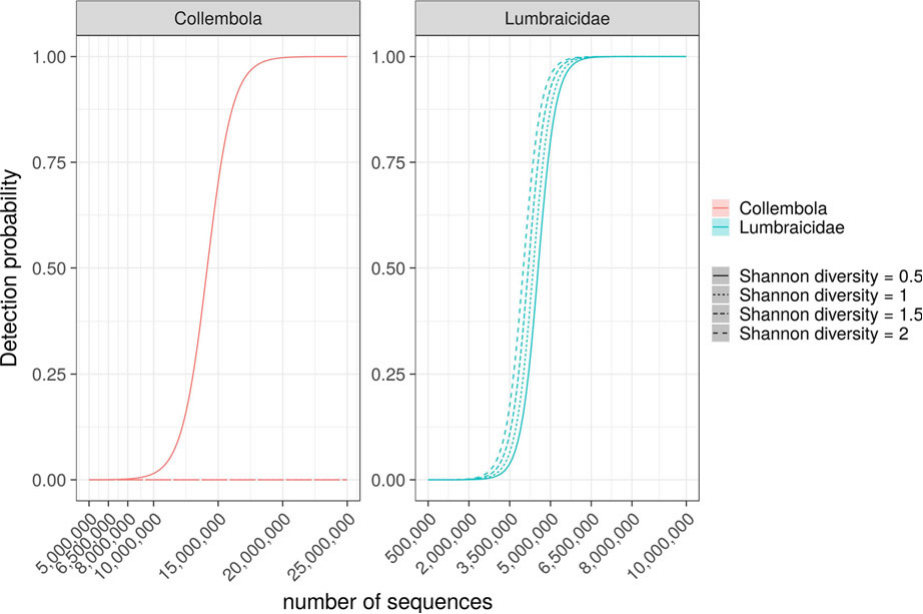
Probability of detecting collembolan and earthworm prey DNA (*y*‐axis) modelled against the number of reads sequenced for each sample (*x*‐axis) using NGS. Only samples that were confirmed to contain DNA of either prey group were used. Line types show how this can be influenced by the diversity of DNA types contained within a sample. Note that these figures do not aim to show the general level of sequencing needed for detection of prey DNA, but to show how sequencing can influence detection probability given the amount of DNA of the respective prey type that was contained in each tested regurgitate sample [Colour figure can be viewed at wileyonlinelibrary.com]

Among nonconsumer species, several species of mammals, a bird, a grass snake and a fish, that are unlikely to belong to the diet of these beetles, were detected in the NGS metabarcoding data (Figure 1a). Furthermore, even though the species detected with NGS from within each diet sample included several taxonomic groups that were not targeted by the multiplex PCR assay (Figure 1a), the species amplified with multiplex PCR were less frequently detected using NGS (Figure 1b, *p* < 0.001). From among the groups targeted by the multiplex PCR, only Collembola that were detected in 58% of all replicates, *R. padi* (9%), Lumbricidae (7%) and *M. dirhodum* (2%), were found among returned reads. In two cases Lumbricidae sequences, and in two cases Collembola sequences, were detected in a regurgitate using NGS, where these were not detected in the same sample by multiplex PCR. Among the samples that were known from NGS and multiplex PCR to contain DNA of the respective targeted prey group, the likelihood of detecting Lumbricidae and Collembola increased with sequencing depth (Figure 3, *p*_Lumbricidae_ < 0.001, *p*_Collembola_ < 0.01). However, the higher the diversity of diet items contained within a sample, the less likely it was to detect these taxa even with a higher sequencing depth (Figure 3, *p*_Earthworm_ < 0.001, *p*_Collembola_ < 0.001). For other prey groups targeted by the multiplex PCR assay, NGS detections were too inconsistent to model this relationship.

**Figure 4:**
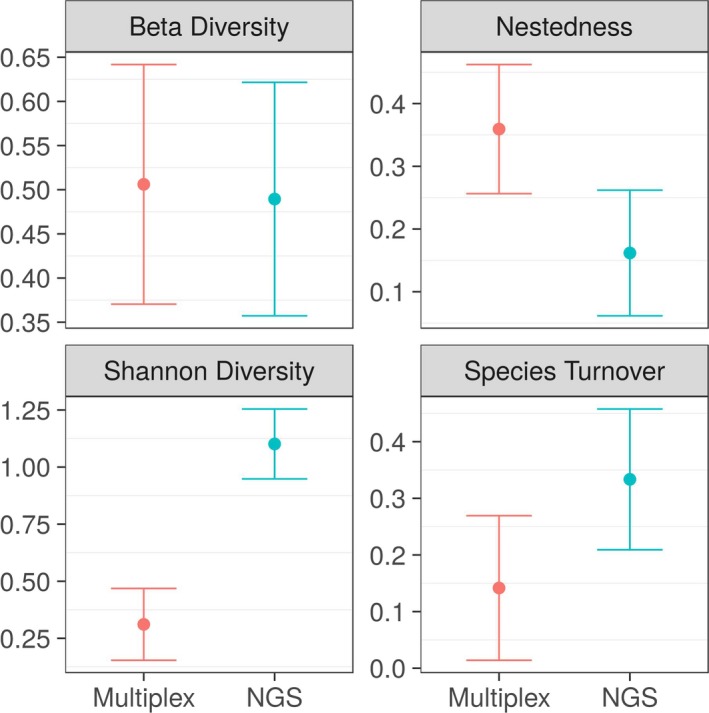
Differences in Shannon diversity and beta diversity of molecularly detected prey taxa between NGS and multiplex PCR in carabid and staphylinid regurgitate samples. “Species turnover” and “nestedness” show the proportion of the beta diversity that is attributable to a change in species detected from replicates of the same sample (species turnover) in contrast to how nested the detection of prey species are. Error‐bars represent 95% confidence intervals [Colour figure can be viewed at wileyonlinelibrary.com]

As expected, the amount of information described on the content of each regurgitate diet sample was considerably greater with NGS than multiplex PCR (Figure 4; Shannon diversity, *p* < 0.001). This information did vary between replicates of the same sample, but the compositional difference between replicates was not different between multiplex PCR and NGS (Figure 4; beta diversity). For NGS, a considerably larger proportion of the beta diversity was, however, attributable to a turnover in the species detected, than with multiplex PCR (Figure 4; species turnover, *p* < 0.05) for which the detection of targeted species was considerably more nested (Figure 4; nestedness, *p* < 0.01).

## 4 DISCUSSION

We here show that NGS metabarcoding of regurgitate samples is more suited to discover the absolute range of interaction partners of species when detailed a priori information on the consumers’ diet is not available, than for comparing the difference between sets of samples. Our results also demonstrate that the sequencing depth needed to describe the diet from samples without preventing the amplification of consumer DNA can be very high (>1,000,000 sequences/sample). This number will, however, differ depending on which metabarcoding primers and sample type is used. With the primers used in this study, regurgitate samples still produced around 90% consumer reads for both tested predator species even when we expected them to be low in consumer DNA (Raso et al., 2014). Blocking primers could reduce this number; however, these would in many cases be problematic to design. Thus, when no method is available that allows NGS‐based diet reads to be increased without increasing sequencing depth, diagnostic PCR would in comparison be more reliable and cost‐effective. This does require that prior knowledge of the likely diet of predators exists. If this is the case and as long as the diagnostic PCR is well designed (Sint et al., 2012), diagnostic PCR will more consistently detect DNA from targeted prey taxa and be less affected by unbalanced DNA concentrations that can bias NGS results. Biases do occur also with diagnostic PCR; however, in a well‐designed detection system, these mostly occur when the measured signal strength (RFU) approaches the defined threshold of detection. Thus, such biases could be compensated for by posteriorly conducting repeated PCRs for samples with (too) low RFU values.

### 4.1 Influence of consumer DNA

As an alternative and more general method to using less general primers or blocking primers (e.g., Vestheim & Jarman, 2008; Piñol et al., 2015; Pompanon et al., 2012), we here wanted to test whether diet samples that are expected to be low in consumer DNA, such as regurgitates, can be metabarcoded without preventing the amplification of consumer DNA (Piñol et al., 2014). By using samples where the proportion of unwanted reads is reduced, this approach could have been used to study predators and prey that are closely related or for examining multiple predator species. However, despite the fact that NGS metabarcoding, even with few diet‐reads, described a greater diversity of the predators’ diet, the returned reads were dominated by the consumer. This caused the diet to be undersampled and highly variable between replicates of the same sample. As the severity of this is likely to vary between samples, depending on their contents and origin, this will make a comparison of samples difficult as prey taxa may be missed by chance. In cases when predator and prey are not closely related (e.g., vertebrates predating on arthropods), this will be less problematic, as less general primers that do not amplify the predator DNA can be used (e.g., Alberdi et al., 2017). However, Alberdi et al. (2017) report similar results to those found here, supporting that when primers that amplify both predator and prey cannot be avoided, consumer reads will be problematic. Paradoxically, this means that even when using primers that in several studies has been reported as superior (more general and less biased; e.g., Elbrecht & Leese, 2015; Rennstam Rubbmark et al., 2018), these may actually bias results more. Mainly because truly unbiased primers (primers that amplify all DNA types equally) may cause diet reads to be swamped by consumer reads.

### 4.2 NGS metabarcoding versus diagnostic multiplex PCR

There was variation between replicates of the same sample for both methods, and with both methods a similar level of compositional variation (beta diversity) in the diet was found. However, with diagnostic PCR this variation mainly occurred when detections were close to the predefined detection threshold of 0.07 RFU as the variability in PCR efficiency sometimes caused samples to test below and sometimes above the threshold. This could be compensated for by testing low RFU value samples repeatedly to confirm the absence of detections (Sint et al., 2011). Furthermore, as diagnostic PCR only detects a defined set of species, this variation only occurs among those species (i.e., it was more nested) and thus is less likely to generate problematic multivariate outliers. This is under the assumption that even if detection of one targeted prey type may be uncertain in an individual sample, others are likely to be more stable. With NGS on the other hand, the beta diversity had a considerably higher turnover component (as new prey species were discovered, others were lost during repeated testing of the same sample), implying that unless a very high number of diet‐reads are generated, NGS will not describe absences of diet items well.

This is doubly problematic as our results suggest that, in addition to already described biases for primers (Alberdi et al., 2017; Leray et al., 2013), differences in proportional concentrations between DNA types (that are likely to vary between samples) strongly influence the sampling completeness in sequencing results. This leads to a situation where sampling effort for a given sample may not only be too low, but vary between sets of samples, and make a comparison of co‐occurring prey difficult between samples (Soberón & Llorente, 1993; Gotelli & Colwell, 2001; Cardoso et al., 2009). Why this occurred is likely because a PCR is a competitive reaction for reagents, where product accumulation generally plateaus as reagents is depleted (Kainz, 2000). If general primers are used, this becomes problematic, especially when initial DNA concentrations are unequal. The reason for this is that this will make the exponential build‐up of products increase higher concentrated DNA types much more rapidly than DNA types with lower concentrations (Kainz, 2000). Ultimately, lower concentration DNA types may be amplified at either a lower rate or not at all (Kainz, 2000).

Another issue with NGS, namely, that as “all” DNA can be detected, what is actually assigned as diet DNA will be an issue of interpretation that may cause included species to be either overestimated or underestimated. This is highlighted by the occurrence of high quality reads from, for example, bird, fish, snake, pig and cow DNA, in beetle regurgitates. The problem is here that while neither method will show if detected diet items were directly consumed by the predator, the presence of nondiet DNA will be more likely in data sets generated with NGS. Mainly because NGS can "detect everything," whereas diagnostic PCR only allow plausible taxa included among tagets to be detected. To compensate for this, it has among other been suggested to filter out low abundance reads (Burgar et al., 2014), or to conduct replicated PCRs and retain shared sequences (De Barba et al., 2014). As shown by the high turnover in species between replicates, here as well as by Alberdi et al. (2017), this will, however, drastically reduce the number of species detected.

### 4.3 When to use each method

Perhaps the primary benefit of diagnostic PCR is that this method will allow the overall detection of certain prey items to be stable even if their DNA is present in different proportions between samples. This requires that one already has some background knowledge on, for example, functionally important players/prey in a system. If such information is not available, we would suggest that NGS could be used to screen the diet of a subset of consumers at either a high sequencing depth or with blocking primers to learn what they eat. Then, from this information, key targets for diagnostic PCR could be selected based on ecological knowledge, in order to reliably capture both the presence and the absence of selected prey targets. For example, the multiplex PCR detection system used for this study was designed to test how the availability of decomposer prey affects the consumption of pest prey in an agricultural setting. This proved to be a well working detection system with targets that were often missed by NGS. This means that depending on whether NGS or diagnostic PCR had been used to investigate the ecological importance of detritivorous prey the importance of these prey would have been judged differently. Among all detections of taxa targeted by diagnostic PCR, we only found two Lumbricidae detections and two Collembola detections in NGS metabarcoding data that were not present for the same sample in diagnostic PCR data. In each case, the detections occurred in only one of the NGS replicates of the sample, and a weak signal was present in the diagnostic PCR data, but this never rose above the defined detection threshold.

In conclusion, we thus suggest that diagnostic multiplex PCR (as long as it has been validated and balanced for amplification strength) is ideally suited for large‐scale screenings of hundreds or thousands of samples as long as taxa that are informative within the framework of the studied questions can be targeted. This information could be supported with NGS metabarcoding data that is more ideally suited to explore unexpected interactions, which, if deemed important then could be included among targeted species to increase the reliability with which those interactions are described.

## Data Availability

Raw sequencing data files are available at NCBI Bioproject: PRJNA498404 and SRA: SRR8109665‐SRR8109770. Multiplex data are available in online supporting information.
