# Supplementary material for: When to use next generation sequencing or diagnostic PCR in diet analyses
Source: Mol Ecol Resour. 2019 Feb 4;19(2):388–99. doi: 10.1111/1755-0998.12974 (PMC6446722; doi:10.1111/1755-0998.12974)
Supplement: Supplementary file 1 [file MEN-19-388-s001.doc]

**Supplement S1; Sample collection**

Predator regurgitates were collected during the spring/summer of 2014 from two agricultural cereal fields located around Kematen in Tirol, Austria for an ongoing study. All predators were collected using dry pitfall traps with wood chips added to provide shelter and minimize in trap predation. To protect from direct sun, debris and rain, a metal roof was installed a few centimeters above each trap. The traps were active for a period of 24 hours, after which all predators were collected alive, placed head first in individual reaction tubes and kept cool until processed further on the same day. To avoid killing of collected beetles and to minimize the amount of consumer DNA in the sample, predators were forced to regurgitate their gut content by a short application of heat stress. After regurgitation the beetle was removed from the tube containing the regurgitate, identified to species (Carabidae) or genus level (Staphylinidae) and later released in the field it originated from. All collected regurgitates were immediately frozen and stored at -80 °C until DNA extraction.
