# Supplementary material for: When to use next generation sequencing or diagnostic PCR in diet analyses
Source: Mol Ecol Resour. 2019 Feb 4;19(2):388–99. doi: 10.1111/1755-0998.12974 (PMC6446722; doi:10.1111/1755-0998.12974)
Supplement: Supplementary file 2 [file MEN-19-388-s002.doc]

**S2; Library preparation protocol**

For library preparation we here used the single tube approach described in Rennstam Rubbmark et al. (submitted). In short, this approach uses universal primers with added non annealing tails to split a single PCR into two steps where (i) a first step of amplification is conducted using the universal primer pair at a higher annealing temperature to obtain higher specificity for the priming site, and (ii) a second step amplification is conducted using the universal tails as templates at lowered annealing temperature. This approach can reduce biases associated with high degrees of degeneracy included in universal primers, minimizes handling and contamination risks, and it is a cost-effective method to include NGS adapters into amplicons for library preparation.

*Protocol*

Using the single tube approach (Rennstam Rubbmark et al. submitted), each PCR was performed in a reaction mix containing 2 µl DNA extract, 0.5 µl of bovine serum albumin (BSA; 10 mg/ml), 0.15 µl of each 1^st^ step primer (10 µM), 1 µl of each 2^nd^ step primer (20 µM), 5 µl of reaction mix (Multiplex PCR Kit Qiagen) and 0.2 µl PCR grade water to adjust the volume to 10 µl*.* Cycling conditions in a Mastercycler Nexus (Eppendorf, Germany) were set to 15 min at 95 °C, 15 step I cycles of 30s at 94 °C, 90s at 55 °C, 60s at 72 °C, and 20 step II cycles of 30s at 94 °C, 90s at 45 °C, 60s at 72 °C, and a final elongation of 10 min at 72 °C.
