# Supplementary material for: When to use next generation sequencing or diagnostic PCR in diet analyses
Source: Mol Ecol Resour. 2019 Feb 4;19(2):388–99. doi: 10.1111/1755-0998.12974 (PMC6446722; doi:10.1111/1755-0998.12974)
Supplement: Supplementary file 3 [file MEN-19-388-s003.docx]

**S3; A review of mean reported sequencing depths per samples for diet sequenced on the MiSeq platform**


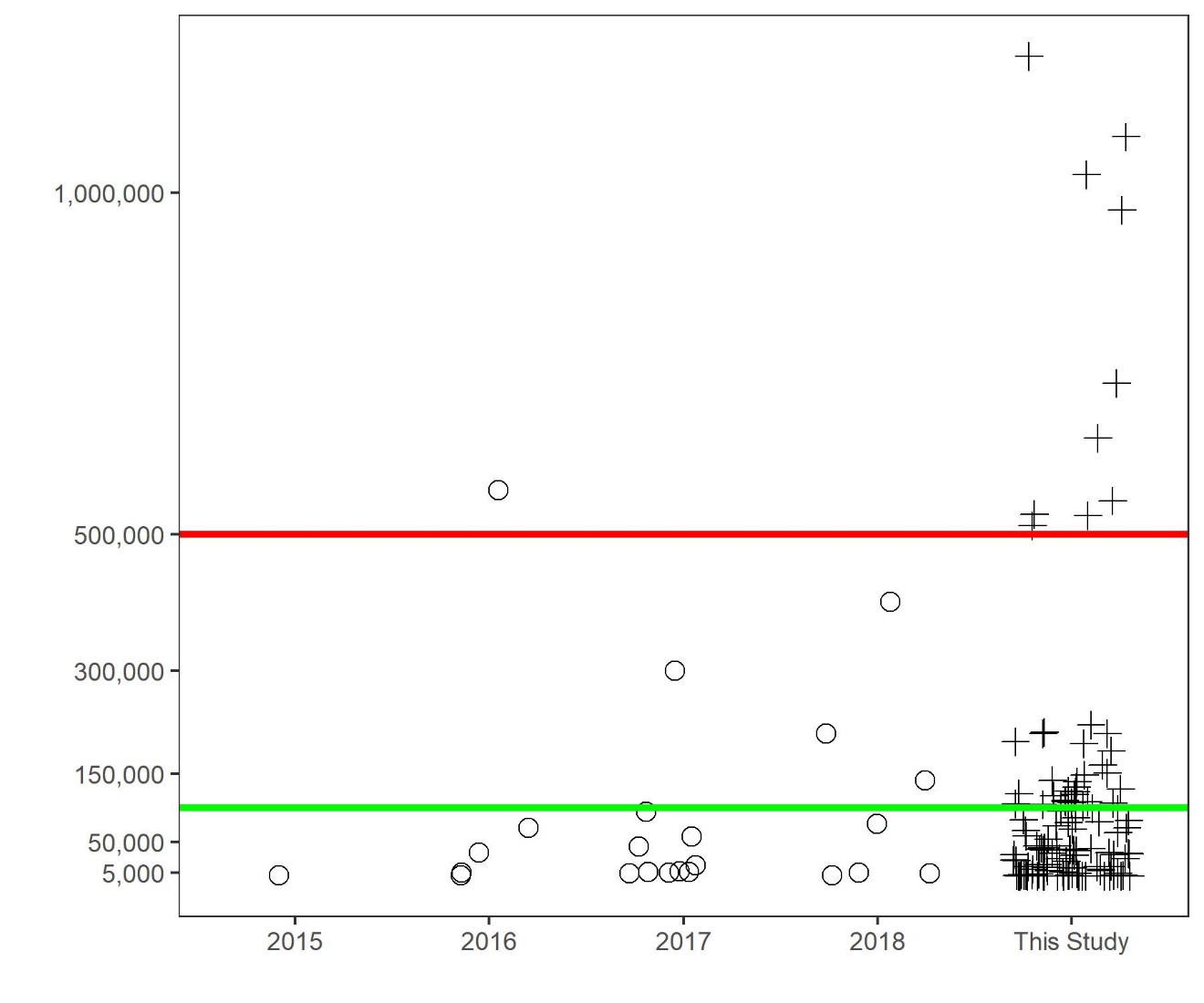


Figure S3.1 Reported mean sequences per samples for diet studies using the MiSeq (Illumina) sequencing platform (circles) split by year. Crosses show the sequencing depth attained per sample in this study. Horizontal lines show the targeted sequencing depth for low (green and high (red) sequencing depth samples in this study.

**Included studies and reported mean sequencing depth per sample**

| Authors | Title | Publication | Volume | Number | Year | Mean sequences / sample |
| --- | --- | --- | --- | --- | --- | --- |
| Thomas, Austen C; Nelson, Benjamin W; Lance, Monique M; Deagle, Bruce E; Trites, Andrew W; | Harbour seals target juvenile salmon of conservation concern | Canadian Journal of Fisheries and Aquatic Sciences | 74 | 6 | 2016 | 1,227 |
| Wang, Wendy Y; Srivathsan, Amrita; Foo, Maosheng; Yamane, Seiki; Meier, Rudolf; | Sorting specimen‐rich invertebrate samples with cost‐effective NGS barcodes: validating a reverse workflow for specimen processing | Molecular Ecology Resources |  |  | 2018 | 1,231 |
| Nakahara, Fumiko; Ando, Haruko; Ito, Hideyuki; Murakami, Asako; Morimoto, Naoki; Yamasaki, Michimasa; Takayanagi, Atsushi; Isagi, Yuji; | The applicability of DNA barcoding for dietary analysis of sika deer | DNA Barcodes | 3 | 1 | 2015 | 1,432 |
| Robeson, Michael S; Khanipov, Kamil; Golovko, George; Wisely, Samantha M; White, Michael D; Bodenchuck, Michael; Smyser, Timothy J; Fofanov, Yuriy; Fierer, Noah; Piaggio, Antoinette J; | Assessing the utility of metabarcoding for diet analyses of the omnivorous wild pig (Sus scrofa) | Ecology and Evolution |  |  | 2018 | 3,857 |
| Jeanniard-du-Dot, Thiphaine; Thomas, Austen C; Cherel, Yves; Trites, AW; Guinet, Chistophe; | Combining hard-part and DNA analyses of scats with biologging and stable isotopes can reveal different diet compositions and feeding strategies within a fur seal population | Marine Ecology Progress Series | 584 |  | 2017 | 3,882 |
| Tiede, Julia; Wemheuer, Bernd; Traugott, Michael; Daniel, Rolf; Tscharntke, Teja; Ebeling, Anne; Scherber, Christoph; | Trophic and non-trophic interactions in a biodiversity experiment assessed by next-generation sequencing | PloS One | 11 | 2 | 2016 | 5,000 |
| Sow, Ahmadou; Brévault, Thierry; Delvare, Gérard; Haran, Julien; Benoit, Laure; d'Acier, Armelle Cœur; Galan, Maxime; Thiaw, Cheikh; Soti, Valérie; Sembène, Mbacké; | DNA sequencing to help identify crop pests and their natural enemies in agro-ecosystems: The case of the millet head miner Heliocheilus albipunctella (Lepidoptera: Noctuidae) in sub-Saharan Africa | Biological Control | 121 |  | 2018 | 5,352 |
| Roswag, Anna; Becker, Nina I; Encarnação, Jorge A; | Isotopic and dietary niches as indicators for resource partitioning in the gleaner bats M. bechsteinii, M. nattereri, and P. auritus | Mammalian Biology |  |  | 2017 | 5,362 |
| Galan, Maxime; Pons, Jean‐Baptiste; Tournayre, Orianne; Pierre, Eric; Leuchtmann, Maxime; Pontier, Dominique; Charbonnel, Nathalie; | Metabarcoding for the parallel identification of several hundred predators and their preys: application to bat species diet analysis | Molecular Ecology Eesources |  |  | 2017 | 5,953 |
| Biffi, Marjorie; Laffaille, Pascal; Jabiol, Jérémy; André, Adrien; Gillet, François; Lamothe, Sylvain; Michaux, Johan R; Buisson, Laëtitia; | Comparison of diet and prey selectivity of the Pyrenean desman and the Eurasian water shrew using next-generation sequencing methods | Mammalian Biology-Zeitschrift für Säugetierkunde | 87 |  | 2017 | 6,775 |
| Danner, Nadja; Keller, Alexander; Härtel, Stephan; Steffan-Dewenter, Ingolf; | Honey bee foraging ecology: Season but not landscape diversity shapes the amount and diversity of collected pollen | PloS One | 12 | 8 | 2017 | 15,936 |
| Mata, Vanessa A; Amorim, Francisco; Corley, Martin FV; McCracken, Gary F; Rebelo, Hugo; Beja, Pedro; | Female dietary bias towards large migratory moths in the European free-tailed bat (Tadarida teniotis) | Biology Letters | 12 | 3 | 2016 | 33,847 |
| Olmos-Pérez, Lorena; Roura, Álvaro; Pierce, Graham J; Boyer, Stéphane; González, Ángel F; | Diet composition and variability of wild octopus vulgaris and alloteuthis media (Cephalopoda) Paralarvae: a metagenomic approach | Frontiers in Physiology | 8 |  | 2017 | 42,963 |
| Krehenwinkel, Henrik; Kennedy, Susan; Pekár, Stano; Gillespie, Rosemary G; | A cost‐efficient and simple protocol to enrich prey DNA from extractions of predatory arthropods for large‐scale gut content analysis by Illumina sequencing | Methods in Ecology and Evolution | 8 | 1 | 2017 | 57,947 |
| Jevit, Matthew J; Janecka, Jan E; Hussain, Shafqat; Muhammad, Ghulam; | Dietary analysis of snow leopards (Panther uncia) in the Gilget-‐Baltistan region of Pakistan using next-‐generation sequencing | |  |  | 2016 | 70,239 |
| Aizpurua, Ostaizka; Budinski, Ivana; Georgiakakis, Panagiotis; Gopalakrishnan, Shyam; Ibañez, Carlos; Mata, Vanessa; Rebelo, Hugo; Russo, Danilo; Szodoray‐Parádi, Farkas; Zhelyazkova, Violeta; | Agriculture shapes the trophic niche of a bat preying on multiple pest arthropods across Europe: Evidence from DNA metabarcoding | Molecular Ecology | 27 | 3 | 2018 | 76,666 |
| Titulaer, Mieke; Melgoza-Castillo, Alicia; Panjabi, Arvind O; Sanchez-Flores, Alejandro; Martínez-Guerrero, José Hugo; Macías-Duarte, Alberto; Fernandez, Jesús A; | Molecular analysis of stomach contents reveals important grass seeds in the winter diet of Baird's and Grasshopper sparrows, two declining grassland bird species | PloS One | 12 | 12 | 2017 | 93,802 |
| Divoll, Timothy J; Brown, Veronica A; Kinne, Jeff; McCracken, Gary F; O'Keefe, Joy M; | Disparities in second‐generation DNA metabarcoding results exposed with accessible and repeatable workflows | Molecular Ecology Resources |  |  | 2018 | 20,8695 |
| Liu, Gang; Shafer, Aaron BA; Hu, Xiaolong; Li, Linhai; Ning, Yu; Gong, Minghao; Cui, Lijuan; Li, Huixin; Hu, Defu; Qi, Lei; | Meta‐barcoding insights into the spatial and temporal dietary patterns of the threatened Asian Great Bustard (Otis tarda dybowskii) with potential implications for diverging migratory strategies | Ecology and Evolution | 8 | 3 | 2018 | 40,1041 |
| Emami-Khoyi, Arsalan; Hartley, David A; Paterson, Adrian M; Boren, Laura J; Cruickshank, Robert H; Ross, James G; Murphy, Elaine C; Else, Terry-Ann; | Identifying prey items from New Zealand fur seal (Arctocephalus forsteri) faeces using massive parallel sequencing | Conservation Genetics Resources | 8 | 3 | 2016 | 563,890 |
| Briem, Felix; Zeisler, Christiane; Guenay, Yasemin; Staudacher, Karin; Vogt, Heidrun; Traugott, Michael; | Identifying plant DNA in the sponging–feeding insect pest Drosophila suzukii | Journal of Pest Science |  |  | 2018 | 140,000 |
| McInnes, Julie C; Alderman, Rachael; Deagle, Bruce E; Lea, Mary‐Anne; Raymond, Ben; Jarman, Simon N; | Optimised scat collection protocols for dietary DNA metabarcoding in vertebrates | Methods in Ecology and Evolution | 8 | 2 | 2017 | 300,000 |
| Berry, Tina E; Osterrieder, Sylvia K; Murray, Dáithí C; Coghlan, Megan L; Richardson, Anthony J; Grealy, Alicia K; Stat, Michael; Bejder, Lars; Bunce, Michael; | DNA metabarcoding for diet analysis and biodiversity: A case study using the endangered Australian sea lion (Neophoca cinerea) | Ecology and evolution | 7 | 14 | 2017 | 5,500 |
